# Does Customized Handle Toothbrush Influence Dental Plaque Removal in Children with Down Syndrome? A Randomized Controlled Trial

**DOI:** 10.3390/healthcare9091130

**Published:** 2021-08-30

**Authors:** Line Droubi, Mohannad Laflouf, Saleh Alkurdi, Salvatore Sauro, Davide Mancino, Youssef Haikel, Naji Kharouf

**Affiliations:** 1 Department of Pediatric Dentistry, Faculty of Dental Medicine, Damascus University, Damascus 0100, Syria; droubiline@gmail.com (L.D.); dr.laflouf@hotmail.com (M.L.); salekh1889@gmail.com (S.A.); 2 Department of Therapeutic Dentistry, I. M. Sechenov First Moscow State Medical University, 119146 Moscow, Russia; salvatore.sauro@uchceu.es; 3 Dental Biomaterials and Minimally Invasive Dentistry, Department of Dentistry, CEU University, 46115 Valencia, Spain; 4 Department of Endodontics, Faculty of Dental Medicine, University Hospital of Strasbourg, 67000 Strasbourg, France; davidemancino@icloud.com (D.M.); youssef.haikel@unistra.fr (Y.H.); 5 Department of Biomaterials and Bioengineering, INSERM UMR_S 1121, Strasbourg University, 67000 Strasbourg, France

**Keywords:** customized handle, dental plaque, toothbrush, down syndrome

## Abstract

The present study aimed to evaluate the effectiveness of customized handle toothbrush in dental plaque removal in children with Down syndrome in comparison to children with no special needs. A randomized clinical trial was performed on 48 children aged 6–9 years old. Participants were divided into two groups (n = 24, children with no special needs or with Down syndrome). Each group was divided into two subgroups (customized and conventional toothbrush). Plaque accumulation was evaluated using Turesky modification of the Quigley–Hein plaque index (TMQHPI) at four times (pre-brushing (T0), post-brushing at baseline (T1), 1 week (T2) and 3 weeks (T3)), and the modified gingival index (MGI) was used to evaluate the gingivitis at three times (T0, T2 and T3). Data were statistically analyzed. Plaque accumulation and gingivitis decreased significantly for all groups between T0 and T3 (*p* < 0.05). Both customized groups demonstrated significant lower plaque accumulation compared to conventional groups (*p* < 0.05) at T1, T2 and T3 and significant lower gingivitis at T3. Customizing the toothbrush handle improved child’s ability for plaque control, especially in children with Down syndrome.

## 1. Introduction

Healthy oral environment is considered a major factor in the prevention of oral diseases and it is an important aspect for the quality of life [1]. Good oral hygiene is essential for preventing dental caries and periodontal diseases; it has been evident that effective daily removal of plaque biofilms plays a major role in maintaining oral health condition [2]. Since plaque is the primary etiological factor in gingivitis and periodontal diseases, effective plaque control can essentially prevent such diseases [3,4]. Although there are several methods for controlling dental plaque, mechanical plaque removal by using a manual toothbrush remains the most effective and reliable method to achieve good oral health [5,6]. In addition, the use of interdental cleaning aids such as dental floss requires repetitive and time-consuming [7], and chemical methods help to improve plaque removal but it is not sufficient without tooth brushing [8]. Manual tooth brushing can be highly effective in plaque removal if performed in a correct manner, based on an adequate technique and time of use [5,6].

Tooth brushing can be difficult in children since it requires manual dexterity, which is not fully developed until they are at least 8 years old [9,10]. The ability of children to perform a more effective tooth brushing increases with age, although parents must be responsible for monitoring their children’s tooth brushing activity until they are 10 years old [11]. Accordingly, it is desirable to use toothbrushes with small head, which corresponds to the size of the children’s mouth, with soft rounded bristles and with short handle and large diameter to fit the child’s hand [12,13]. It is highly recommended to have a design and length that properly fit the child’s hand to improve the quality of tooth brushing [14].

Down syndrome (DS) is one of the most leading causes of disability; millions of these patients face various health issues. The incidence of DS ranges from 1 in 319 to 1000 people worldwide [15]. DS phenotype is caused by a complete or partial trisomy (TS21) of human chromosome 21 (HSA21) acquired essentially by meiotic non-disjunction events during gametogenesis [16]. Individuals with DS have specific orofacial characteristics associated with such a syndrome, for instance periodontal disease, malocclusion, mouth breathing, macroglossia, delayed teeth eruption, missing and malformed teeth, and microdontia [17,18,19]. Manual dexterity difficulties may lead to poor oral hygiene, which in turn compromises the effectiveness of their tooth brushing practices, and may result in accumulation of plaque and debris, hence favoring development of periodontal diseases [19,20]. These difficulties are due to debilitating musculoskeletal problems [21]. However, manual dexterity in DS can be improved with training and support [22]. There are different ways to improve tooth brushing in children with DS such as electric toothbrushes or modified handle toothbrushes [23].

Various toothbrush adaptations were described to adjust toothbrush handles, some of these modifications on manual toothbrush handles were: enlarged handles, handles with an elastic cuff, bicycle handlebar grips, tennis ball handles, handles with attached strap and customized handles [23,24]. These modifications were studied in patients with poor fine motor skills, restricted hand and finger movements [25] and the elderly [26].

Nevertheless, in the literature, there is very little information on plaque control using customized handle toothbrushes among children with Down syndrome.

Thus, the aim of this study was to evaluate the effectiveness of customized handle toothbrush in dental plaque control in children with DS and children with no special needs. The null hypothesis tested was that the customized handle toothbrush would have no effect on accumulation of plaque among children with Down syndrome and/or children with no special needs.

## 2. Materials and Methods

### 2.1. Study Design

This study is a parallel-arm, double-blind, randomized clinical trial studying the effectiveness of customized handle toothbrush in dental plaque control in children with no special needs and children with Down syndrome.

The study was conducted in a governmental charity and special needs charity (Damascus, Syria) between April 2021 and May 2021. The study adhered to the ethical values of the Declaration of Helsinki and was approved by the Ethic Committee at the Ministry of Higher Education in Syria (1674/SM). All participants’ parents were informed of the study’s procedure and objectives and were included only after providing informed written consent. To ensure the quality and transparency of this randomized clinical trial, the authors followed the CONSORT statement [27,28]. This RCT is registered in the Clinical Trials database (NTC04845633).

### 2.2. Sample Size Calculation

Sample size was calculated using G*Power 3.1.9.2 software (Heinrich-Heine-Universität Düsseldorf, Düsseldorf, Germany). Four subgroups of 12 participants each were finally formed in order to have 95% power and an alpha error probability of 0.05.

### 2.3. Randomization and Blinding

Randomization was accomplished using an online software at www.randomizer.org (accessed on 11 April 2021) in order to allocate children with no special needs and children with down syndromes into two groups (conventional toothbrush and/or customized handle toothbrush). Block randomization method was performed to get equal and balanced groups. First of all, participants were assigned either into healthy or Down syndrome participants. Then, they were given numbers and allocated into equal groups with www.randomizer.org.

This study was a double-blind study. Blinding was achieved according to the following methods: assessment was performed by two professional investigators for whom assessment methodology was explained and they were blinded on outcomes by not knowing the type of toothbrush used for the participant. Data analyzer blinding was achieved by encoding the results of each toothbrush with a special code so that the type of toothbrush could not be identified

### 2.4. Participants

Children with no special needs participants were recruited from children residing in a governmental charity in Damascus, Syria, and children with Down syndrome were recruited from special needs charity in Damascus, Syria.

All participants were recruited fulfilling the inclusion criteria: aged 6–9 years old, the presence of at least 10 teeth free from dental caries on both buccal and lingual surfaces of the teeth, Turesky modification of the Quigley–Hein plaque index (TMQHPI) is at least 2, cooperative participant (Positive according to Frankel’s behavioral rating scale), and participant with Down syndrome must have been diagnosed by a specialist medical operator and after obtaining parents’ consent. The exclusion criteria were: children undergoing orthodontic treatment, allergic to any of the toothpaste ingredients used in the study, children with no special needs participant with systemic diseases, and children with Down syndrome who suffer from chronic weakness such as epilepsy or taking medications continuously.

The participating children of each group were divided randomly into two subgroups (n = 12) as following:

Group 1 (children with no special needs):

Subgroup A (SubA): Children with no special needs who used a conventional toothbrush.

Subgroup B (SubB): Children with no special needs who used a customized handle toothbrush.

Group 2 (children with Down syndrome):

Subgroup C (SubC): Children with Down syndrome who used a conventional toothbrush.

Subgroup D (SubD): Children with Down syndrome who used a customized handle toothbrush.

### 2.5. Intervention and Customized Handle Toothbrush Fabrication

The modified Stillman tooth brushing technique [29] was shown to the parents and children using models and their compliance to the instructions were checked by asking them to demonstrate the same. All participants were provided with Vitis^®^ Junior toothbrush (nylon, rounded tip and soft bristles) (Vitis^®^, Barcelona, Spain). All the participants were instructed to brush twice daily (morning and night) for 3 min and to use Vitis^®^ Junior gel toothpaste (Vitis, Spain) during the study period. A pea-sized amount of toothpaste was instructed to be used by the participants and finally rinse with water after tooth brushing. Monitoring of tooth brushing among the children was performed by parents for the entire period of the study. Participants also consented to refrain from brushing their teeth and performing any other oral hygiene procedures for 24 h prior to the start of the study.

**Figure 1:**
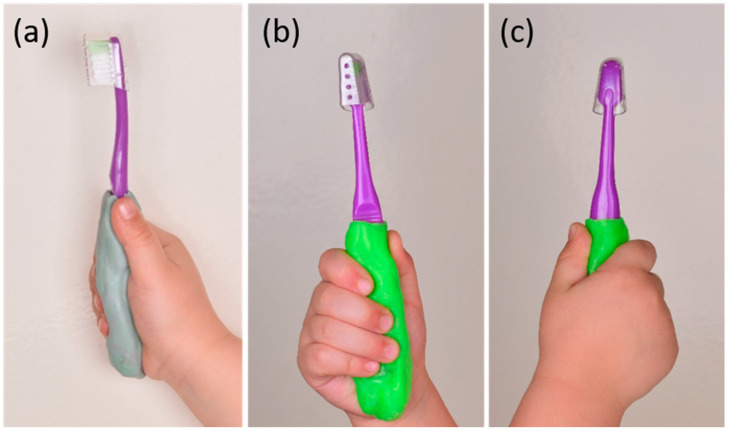
Fabrication of toothbrush handle: (**a**) Molding of participant’s hand condensation silicone; (**b**,**c**) Three-dimensional printed customized handle for toothbrush using Poly Lactic Acid material.

Customized handle toothbrush for the children in SubB and SubD were fabricated using Polylactic acid material (Zhejiang Flashforge 3D Technology Co., Ltd.; Zhejiang, China). A three-dimensional printer (Dedibot DF3 3D Printer; Hangzhou DediBot Intelligent Technology Co., Ltd., Hangzhou, China) was used to fabricate the customized handle toothbrush, after taking a mold of a child’s grip with rubbery material (Poly Siloxane putty Zhermack, Badia Polesine, Italy) (Figure 1) [30].

### 2.6. Evaluation and Follow Up

Plaque accumulation was measured using the TMQHPI index [31,32] by applying disclosing solution (“Mira-2-Ton”, Hager Werken, Duisburg, Germany). Through this method, the disclosed plaque of all primary and permanent teeth (except surfaces with restorations and malformations) was scored using a 0–5 scale. Mean plaque scores were obtained for each subject at each examination by totaling the individual plaque scores and dividing that sum by the number of gradable sites examined. A grading system based on the criteria proposed by Turesky et al. [32] (Table 1) was applied to evaluate the plaque formation. Plaque accumulation was evaluated at four times (pre-brushing (T0), post-brushing at baseline (T1), 1 week (T2) and 3 weeks (T3)). Two experienced pediatric examiners analyzed dependently the plaque accumulation by using TMQHPI index. When different scores were attributed by the two examiners, they reanalyzed the micrograph with a third examiner to reach an agreement. Micrographs, showing the assessment of TMQHPI, were taken at each period and for each group using a camera (Nikon D5300, Tokyo, Japan).

In addition to TMQHPI index, gingivitis was measured by the Modified Gingival Index (MGI) [33] at three periods {pre-brushing at baseline (T0, 1 week (T2) and 3 weeks (T3)}. Gum status was evaluated using a score ranging between 0 and 4 (Table 2). Mean gingival scores were obtained for each subject at each examination by totaling the individual gingival scores and dividing that sum by the number of gradable sites examined.

### 2.7. Statistical Analysis

Cronbach’s alpha test was applied to verify the reliability between the two observers using SPSS 23 package. Statistical Analysis was performed using the SPSS program version (SPSS Inc., Chicago, IL, USA). The Kolmogorov–Smirnov test used to determine the data distribution for (T0) values revealed an abnormal distribution of the data; therefore, nonparametric tests were used for the statistical analysis of this outcome. Thus, the Kruskal–Wallis test was used including a multiple comparison procedure (Mann–Whitney U and Wilcoxon tests). A significance level at α = 0.05 was adopted.

## 3. Results

**Figure 2:**
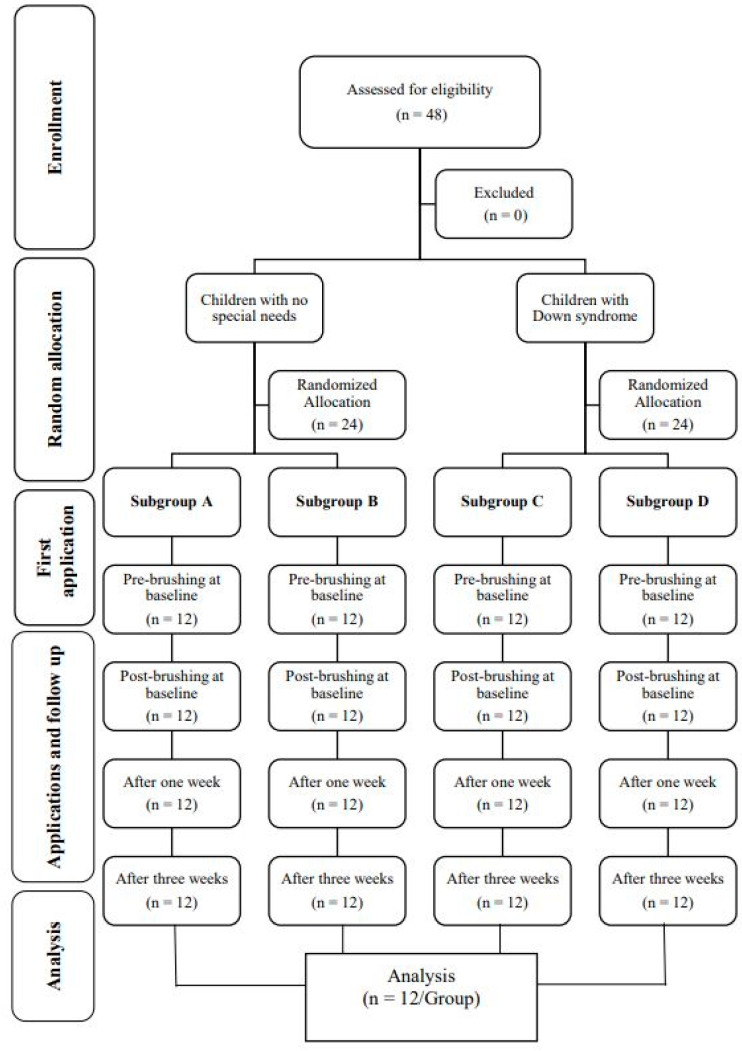
CONSORT flow chart of the trial during the levels of the study and follow up.

Participant flow through the study is illustrated in the Consort flow diagram shown in Figure 2.

Forty-eight participants were enrolled in this study, 26 females and 22 males. Means and standard deviations of TMQHPI scores are described in Table 3. The Cronbach’s alpha value for interobserver agreement of all subgroups was 0.993, which figures an excellent reliability between the observers. 

Both subgroups of children with no special needs showed lower plaque accumulation scores than DS subgroups at T0 (*p* < 0.05).

At T1, no significant difference (*p* > 0.05) was observed regarding the amount of plaque accumulation between the SubB and SubD groups. Meanwhile, there were a significant difference between SubA and SubC. There was a significant difference between both subgroups of children with no special needs (*p* < 0.05). Concerning the subgroups of DS children, SubD had lower plaque accumulation scores than SubC.

At T2 and T3, There was a significant difference between the subgroups in each main group (*p* < 0.05); between SubA and SubC (*p* < 0.05); and SubB with SubD (*p* < 0.05).

**Figure 3:**
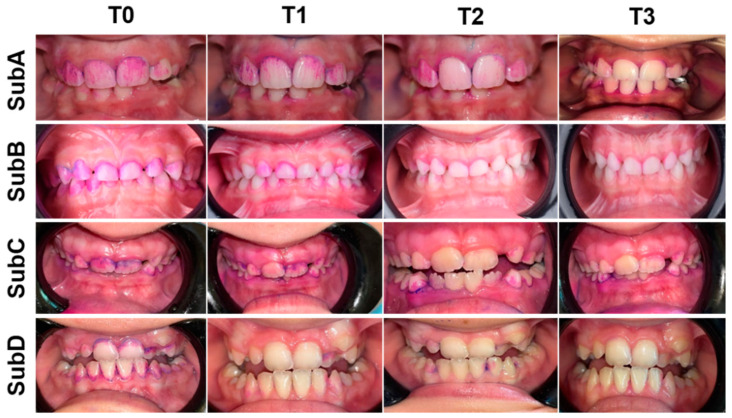
Assessment of (TMQHPI) for conventional/customized handle toothbrush in children with no special needs and children with Down syndrome at the studied time points (pre-brushing (T0), post-brushing at baseline (T1), 1 week (T2) and 3 weeks (T3)).

Each subgroup showed an improvement in plaque accumulation scores in time (T0 to T3) (*p* < 0.05) (Table 2 and Figure 3), except for the two last stages (T2 and T3) of SubA, which presented no significant difference (*p* > 0.05).

Means and standard deviations of gum status for the (MGI) are represented in Table 4. Mann–Whitney U test exposed a significant difference at the baseline between the SubA and SubC groups and between the SubB and SubD groups. Moreover, a significant difference was found at T2 between the SubA and SubC groups and between the SubB and SubD groups. When it came to T3, a significant difference was observed between all each paired groups. Finally, a Wilcoxon test revealed a significant difference for each group at all studied periods, where a considerable improvement of MGI was observed through the study.

## 4. Discussion

The target for maintaining a good oral health would be accomplished only if there was adequate plaque control. Many modifications of handle design were found, such as straight, angled, curved, and contoured with grips and with soft rubber areas to make handles easier to hold, use and control [34,35]. Tooth brushing manual skills were not fully developed in children younger than 8–10 years of age [9,10]. According to Pujar et al. [10], plaque removal efficacy improved with age (57% in 6-year-olds and 82% in 12-year-old children). Hence, toothbrush handle modifications provided assistance for toothbrush in children.

This study aimed to evaluate the effectiveness of customized handle toothbrush in dental plaque control among children with no special needs and children with DS. Plaque control evaluation was performed by applying disclosing solution to all teeth surfaces. This method permits identifying of plaque accumulation and the use of TMQHPI evaluation on buccal and lingual surfaces of all teeth. This plaque index was well suited for registering plaque in children’s teeth [36,37]. This study showed that in children aged 6–9 years old, better plaque control was noticed in the customized handle toothbrush group compared to the conventional one. Therefore, based on the results of this study, the null hypothesis, that the customized handle toothbrush would have no effect on accumulation of plaque, was rejected.

At T0, TMQHPI showed higher plaque amount in children with DS group than for the healthy children group. After monitoring subgroup A and subgroup C in time (T1, T2, T3), it was found that plaque control is better in healthy children at all time periods of evaluation. These findings could be due to better neuromuscular coordination in children with no special needs. Moreover, TMQHPI scores in T2 and T3 for subgroups B and D showed a significant difference, whereas the healthy group was better in plaque control. At T1, no significant difference was found between customized handles subgroups, which may be due to being given brushing instructions only recently.

The MGI index in children with no special needs was significantly lower than that for children with Down syndrome at all periods, regardless of the type of toothbrush used. The study of Morinushi et al. [38] that compered gingivitis in children with Down syndrome with a healthy control group of children found that gingivitis in children with Down syndrome is higher than in healthy children. At T3, significant difference was found between customizing handles subgroup and conventional handle subgroup in children with no special needs and children with Down syndrome. In addition, this study found that all subgroups had a significant decrease in gingivitis at each time.

People with disabilities often need extra help to achieve and maintain good oral health. Although caregiver role in maintaining good oral hygiene can be extremely helpful, it is emphasized if patients can maintain and optimize their own oral heath by self-brushing and cleaning, which can help developing dependency and self-esteem [23].

Using a manual toothbrush with added grip on it can allow patients to have good joints and muscle stimulation. Patients can use electric toothbrushes [39]; however, it does not help in developing manual dexterity [39]. In addition, the electric toothbrush is more expensive than manual ones and the manual ones are handier. In the present study, the customized handle fits the size of the participant’s grip, while the electric toothbrush has a unified grip for everyone, in this case the participant might feel more ownership towards their stuff and take more care of their health. 

In the current study, customized handles were designed to be compatible with a handgrip for participants with DS. The use of customized handle toothbrushes showed significant improvement in daily plaque control in children with DS comparing with to conventional toothbrushes. Many researches have studied the manual dexterity of persons with Down syndrome and found that such persons suffered from difficulties in hand movement and control [40]. Despite such difficulties, it is possible to improve manual dexterity skills by constant training and by external support tools [41]. It has been found that training children on hands movement enhances children’s hands movement control [22].

**Figure 4:**
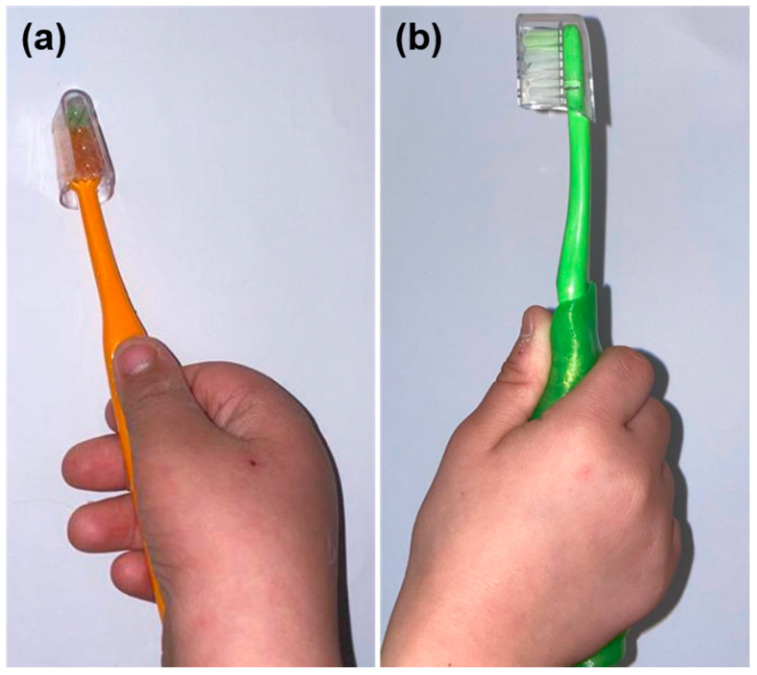
(**a**) Conventional handle toothbrush and (**b**) customized handle toothbrush in children with Down syndrome.

A customized handle helps to facilitate brushing teeth since participants find it easy to hold (Figure 4). This is especially advantageous for those with compromised grasp, such as the elderly, children and patients with developmental disabilities [23,26]. According to the results of this study, it was observed that both techniques helped in improving the child’s plaque control. Furthermore, the customized group maintained a persistent progression in all stages of the study, whereas the conventional one reached its peak after a week of assessment.

Multiple materials have been used to fabricate customized handles such as acrylic resins [26], light-cured composite material [25], silicone putty [42], clay materials [39] and by using 3D printing technology [30]. Although acrylic resins are low cost, its processing time is long. and the laboratory procedures are complicated. Light-polymerized composite material is simple and its required time is short, but the obstacle is the high cost of the composite and the light polymerizing box [26]. The disadvantages of using silicone putty or clay materials are that they have to be replaced with every toothbrush, and their ability to rupture. In this study, a 3D printer was used to produce customized toothbrush handles made from PLA material. This technique is particularly user-friendly to get a rapid, flexible choice to manufacture a toothbrush handle and the possibility of making adjustments. In addition to that, it uses PLA durable material, to serve the participant for a long time, which can be reused with a new toothbrush when it needs to be replaced [30,43]. 

Although several studies have shown significant improvements in participants’ ability to control dental plaque by using customized handle, it is difficult to compare the current study with previous studies that evaluated the effectiveness of customized handle toothbrush in improving dental plaque control due to different sample groups involved in previous studies. Kammers et al. [26], who compared the adapted toothbrush handle with the conventional type in children with no special needs, found that adapted toothbrush handles were more efficient in reducing the level of biofilm on dentures in the elderly. Another study revealed the effectiveness of customized handle by monitoring the difference in oral health evaluation before and after using a toothbrush with customized handle in patients with stroke ischemic [44].

The limitations of this study were that children with special needs such as DS are not as available as children with no special needs; in addition to that, the age range group made it more difficult to find participants. Another limitation was the difficulty to apply blinding concerning the used toothbrush type on the participants involved in this RCT. Furthermore, it was the responsibility of the parents to adhere to the required instructions.

## 5. Conclusions

Customizing the toothbrush handle can maintain and optimize the oral health of children with no special needs and children with Down syndrome by self-brushing and cleaning, which can help develop dependency and self-esteem. Based on the results of the TMQHPI values obtained in this study, it is possible to affirm that the use of a customized handle toothbrush may be more effective in oral plaque control comparing with a conventional toothbrush in both children with no special needs as well as in children with Down syndrome.

## Figures and Tables

**Table 1:** A grading system for plaque accumulation Inspired from Ref. [32] with authorization.

Score	Criteria
0	No plaque present
1	Separate flecks of plaque at the cervical margin
2	A thin continuous back of plaque (up to 1 mm) at the cervical margin
3	A band of plaque wider than 1 mm but covering less than one-third of the side of the crown of the tooth
4	Plaque covering at least one-third but less than two-thirds of the side of crown of the tooth
5	Plaque covering two-thirds or more of the side of the crown of the tooth

**Table 2:** The grading system of gum status for the (MGI) [33].

Score	Criteria
0	Absence of inflammation
1	Mild inflammation or with slight changes in color and texture but not in all portions of gingival marginal or papillary
2	Mild inflammation, such as the preceding criteria, in all portions of gingival marginal or papillary
3	Moderate, bright surface inflammation, erythema, edema and/or hypertrophy of gingival marginal or papillary
4	Severe inflammation: erythema, edema and/or marginal gingival hypertrophy of the unit or spontaneous bleeding, papillary, congestion or ulceration

**Table 3:** Mean and standard deviations for each score of plaque accumulation at the different periods for SubA: Children with no special needs who used a conventional toothbrush; SubB: Children with no special needs who used a customized handle toothbrush; SubC: Children with Down syndrome who used a conventional toothbrush; SubD: Children with Down syndrome who used a customized handle toothbrush. Upper letters (^a–h^) indicate statistically significant differences (raw) between the different subgroups (*p* < 0.05).

Time\Subgroups	SubA	SubB	SubC	SubD	*p* < 0.05
T0	3.19 ± 0.5 ^a^	3.05 ± 0.18 ^c^	3.63 ± 0.29 ^b^	4.41 ± 0.25 ^d^	a < b & c < d
T1	1.7 ± 0.68 ^a,e^	0.96 ± 0.29 ^b^	2.69 ± 0.45 ^c,f^	0.97 ± 0.35 ^d^	a > b & c > d e < f
T2	1.41 ± 0.39 ^a,e^	0.38 ± 0.43 ^b,g^	2.23 ± 0.54 ^c,f^	0.52 ± 0.21 ^d,h^	a > b & c > d e < f & g < h
T3	1.21 ± 0.47 ^a,e^	0.14 ± 0.27 ^b,g^	1.9 ± 0.58 ^c,f^	0.26 ± 0.1 ^d,h^	a > b & c > d e < f & g < h
*p* < 0.05	T0 > T1 T1 > T2	T0 > T1 T1 > T2 T2 > T3	T0 > T1 T1 > T2 T2 > T3	T0 > T1 T1 > T2 T2 > T3	

**Table 4:** Mean and standard deviations for each score of gum status for the (MGI) at the different periods for SubA: Children with no special needs who used a conventional toothbrush; SubB: Children with no special needs who used a customized handle toothbrush; SubC: Children with Down syndrome who used a conventional toothbrush; SubD: Children with Down syndrome who used a customized handle toothbrush. Upper letters (^a–h^) indicate statistically significant differences (raw) between the different subgroups (*p* < 0.05).

Time\Subgroups	SubA	SubB	SubC	SubD	*p* < 0.05
T0	1.4 ± 0.76 ^a^	1.12 ± 0.64 ^c^	2.95 ± 0.72 ^b^	2.91 ± 0.73 ^d^	a < b & c < d
T2	0.8 ± 0.43 ^a,e^	0.41 ± 0.46 ^b,g^	1.95 ± 0.62 ^c,f^	1.41 ± 0.55 ^d,h^	e < f & g < h
T3	0.54 ± 0.49 ^a,e^	0.83 ± 0.19 ^b,g^	1.54 ± 0.62 ^c,f^	0.75 ± 0.45 ^d,h^	a > b & c > d e < f & g < h
*p* < 0.05	T0 > T2 T2 > T3	T0 > T2 T2 > T3	T0 > T2 T2 > T3	T0 > T2 T2 > T3	

## Data Availability

Not applicable.
